# Scholars for Life! Building Faculty and Community Connections on an Age-Friendly University Campus

**DOI:** 10.1093/geroni/igab046.1494

**Published:** 2021-12-17

**Authors:** Andrea June, Carrie Andreoletti

**Affiliations:** 1 Central Connecticut State University, Central Connecticut State University, Connecticut, United States; 2 Central Connecticut State University, New Britain, Connecticut, United States

## Abstract

Central Connecticut State University’s Scholars for Life! supports the engagement of older learners in the community through faculty guest lectures. During the COVID-19 pandemic, participation in the virtual format frequently swelled to over 100 attendees, which is five times the number participating pre-pandemic. Moreover, faculty engagement increased. This presentation will share results of a study that used an Age-Friendly University (AFU) lens to explore this expanded connection to community members with the intention to build on its successful faculty-community engagement. 132 participants responded to the survey (M age = 69), mostly identifying as local retired alumni and community members. Participants reported high satisfaction with the lectures, connection to the university, interest in joining future travel abroad experiences, and utilizing campus resources when safe. Indeed, 84% are now aware of CCSU’s AFU status and 61% expressed interest in the 62+ course tuition waiver. Implications and future directions will be addressed.

